# Scale-Sensitive Feature Reassembly Network for Pedestrian Detection

**DOI:** 10.3390/s21124189

**Published:** 2021-06-18

**Authors:** Xiaoting Yang, Qiong Liu

**Affiliations:** School of Software Engineering, South China University of Technology, Guangzhou 510006, China; 201820137807@mail.scut.edu.cn

**Keywords:** pedestrian detection, scale variation, feature fusion, RoI feature, road scene

## Abstract

Serious scale variation is a key challenge in pedestrian detection. Most works typically employ a feature pyramid network to detect objects at diverse scales. Such a method suffers from information loss during channel unification. Inadequate sampling of the backbone network also affects the power of pyramidal features. Moreover, an arbitrary RoI (region of interest) allocation scheme of these detectors incurs coarse RoI representation, which becomes worse under the dilemma of small pedestrian relative scale (PRS). In this paper, we propose a novel scale-sensitive feature reassembly network (SSNet) for pedestrian detection in road scenes. Specifically, a multi-parallel branch sampling module is devised with flexible receptive fields and an adjustable anchor stride to improve the sensitivity to pedestrians imaged at multiple scales. Meanwhile, a context enhancement fusion module is also proposed to alleviate information loss by injecting various spatial context information into the original features. For more accurate prediction, an adaptive reassembly strategy is designed to obtain recognizable RoI features in the proposal refinement stage. Extensive experiments are conducted on CityPersons and Caltech datasets to demonstrate the effectiveness of our method. The detection results show that our SSNet surpasses the baseline method significantly by integrating lightweight modules and achieves competitive performance with other methods without bells and whistles.

## 1. Introduction

Pedestrian detection aims to predict the position coordinates of all pedestrian instances in images or videos. It is a critical problem in computer vision field with many real-world applications, such as autonomous driving, intelligent surveillance, and robotics. Furthermore, in academic fields, pedestrian detection is also a fundamental component for research hotspots, including person search [1], object tracking [2], and human pose estimation [3,4].

**Figure 1:**
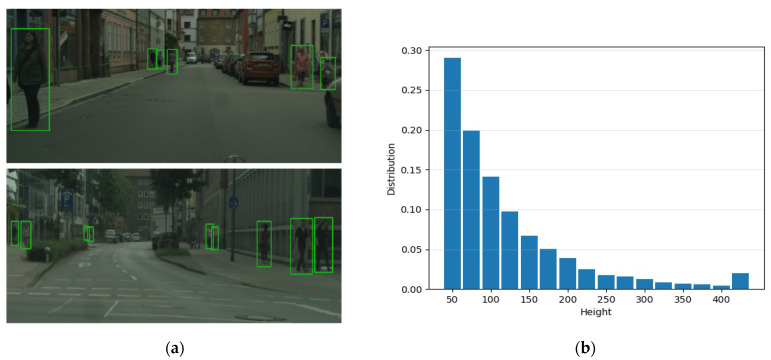
The elaboration of pedestrian scale variation in the CityPersons dataset. (**a**) Some example pedestrian images in road scenes; (**b**) the distribution of pedestrian heights in the CityPersons training set. One can observe that small-size (i.e., small height) instances indeed dominate the distribution.

Recently, with the rapid development of deep convolutional neural networks (CNNs), CNN-based detectors have become the dominant trend in pedestrian detection. Although state-of-the-art approaches have achieved remarkable progress on standard pedestrians, they still suffer from scale variation. As shown in Figure 1a, the images captured in road scenes usually contain a variety of pedestrians, and more pedestrians are of a small scale. We further analyze the pedestrian height distribution on a CityPersons [5] training set in Figure 1b. Statistically, the scales of pedestrian instances distribute in a wide range, i.e., from 1 pixel to 963 pixels, and about 63% of the instances have a height smaller than 100 pixels, which leaves a critical bottleneck for detection performance. Due to the blurred appearance of small-scale pedestrians, it is hard to distinguish them from the background and other overlapped pedestrians, which will result in missed detections. Meanwhile, large-scale pedestrians typically exhibit dramatically different characteristic representations from the small ones, which presents an extreme challenge to the scale-invariant ability of CNNs.

**Figure 2:**
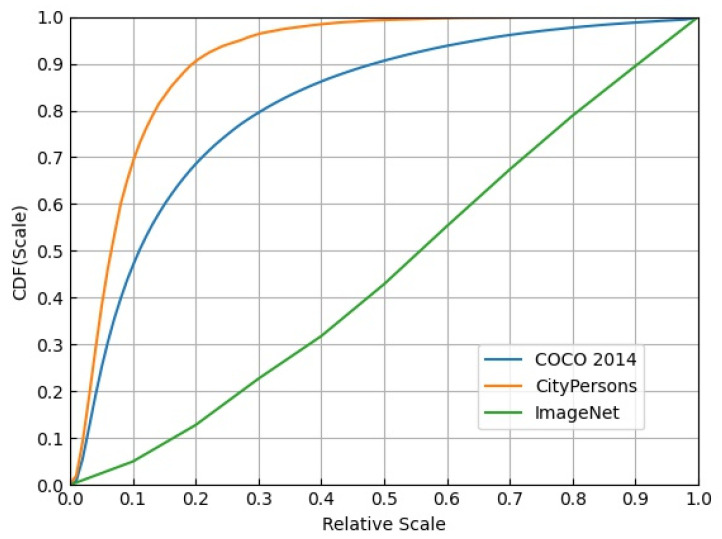
Fraction of objects in the dataset vs. scale of objects relative to the image. The x-axis indicates the ratio of the area of a single object to that of the entire image. The y-axis is the value of the cumulative distribution function (CDF), which indicates the percentage of the cumulative number of objects with the same relative scale to all objects.

The small pedestrian relative scale (PRS) makes the problem even worse. Figure 2 shows the scale of objects relative to the image in ImageNet [6], COCO [7], and CityPersons datasets. The orange curve representing the CityPersons dataset is the steepest among the three curves, and over 70% of instances occupy less than 1% of the whole image area. Furthermore, the median relative scale of instances in CityPersons is 0.065, which is the smallest compared with that of the ImageNet and COCO (0.556 and 0.110, respectively). Thus, the detector is incapable of effectively extracting RoI (region of interest) features under such an extreme condition.

To tackle these scale challenges, an intuitive way is using image pyramids [8,9], which take multiple images as the input by scaling or cropping the original image to several scales. However, because such an approach does not share the extracted features, it suffers from unaffordable computations. Another line of research [10,11,12,13,14] focuses on in-network feature pyramids rather than out-network image pyramids. FPN [10] is a representative method. It employs a top-down pathway with lateral connections to fusion multilevel features, which significantly improves the detection performance. Nevertheless, the backbone network (e.g., ResNet [15]) that supplies the multilevel features for FPN has some intrinsic flaws. Specifically, a larger sampling stride of the high-level feature map is prone to skip smaller pedestrians directly, resulting in the inadequate sampling of small instances. Moreover, the receptive field of the convolutional layer is fixed and lacks the adaptability to pedestrians of different scales, while [16] proved that the most suitable receptive field is strongly related to object scales. In most methods, these flaws are not considered simultaneously.

In the conventional FPN architecture, high-level features are gradually fused with adjacent feature layers to countervail the scarceness of semantics in low-level features. In this paradigm, the semantic information of the top-level feature is critically important. However, in order to reduce computational overhead, these approaches usually employ a 1×1 convolutional layer to compress the features in channel dimensions, making top-level features suffer from semantic information loss. 

The fused feature pyramid is further exploited to extract RoI features and perform proposal refinement. In this stage, the traditional strategy heuristically assigns each RoI to a specific feature level according to its scale. As mentioned above, it is difficult to obtain high-quality RoI features in the case of small PRS, while such a heuristic scheme ignores the potentially beneficial information from other feature levels, making this issue more challenging. Taking this into account, PANet [11] pools each RoI in all feature layers and further aggregates them for the predictions that follow. However, its max fusion operation may discard useful information with low response, and the extra fully connected layers used to adapt multiple RoI features greatly increase the computational overhead of the model.

To summarize, for scale challenges in pedestrian detection, there are three problems with current methods:

- The inadequate sampling of the backbone network caused by a larger stride and fixed receptive field impedes the downstream feature pyramid fusion, and these two flaws are not considered simultaneously in most methods.
- The high-level feature map faces semantic information loss during feature compressing in channel dimensions, while this feature is critically important in a top-down propagation pathway.
- The traditional assignment strategy that relies on the scales of proposals cannot cope with the dilemma of small PRS, resulting in coarse RoI representation, which is unfavorable to both classification and regression tasks.

In this paper, a scale-sensitive feature reassembly network (SSNet) is proposed for pedestrian detection with high accuracy and affordable computation, which is built on the Faster R-CNN model [17]. Specifically, our SSNet includes three novel modules to address the above problem, respectively, and they are used in different stages of the detection pipeline. First, a multiparallel branch sampling (MBS) module is embedded in the backbone network to achieve better sampling. Inspired by previous efforts [18,19], it consists of two parts: a flexible anchor stride adjustment scheme for capturing more small samples and a modified RFB [19] block for generating multiscale features. Subsequently, a context enhancement fusion (CEF) module is devised to mitigate the information loss of high-level features in the channel unification of FPN [10]. Finally, an RoI feature reassembly (RFR) module is adopted to refine the proposal representations. Different from the heuristic strategy, each RoI is assigned to all feature levels in RFR, and the RoI features are reassembled based on the contributions of multiple feature levels. As a result, our SSNet outperforms the baseline method significantly and achieves competitive accuracy with some state-of-the-art methods.

The contributions of this paper are summarized as follows:

1. A novel multi-parallel branch sampling module is designed to remedy inadequate sampling of the backbone network. By flexibly adjusting the receptive field and anchor stride, we extract scale-sensitive features with a uniform representational power.
2. A context enhancement fusion module is devised to inject various spatial context information into the original features. It notably reduces the information loss of high-level features in channel unification.
3. An RoI feature reassembly module is introduced to cope with the dilemma of small PRS. Each RoI feature is reassembled based on the contributions of different feature levels, which is beneficial to both classification and regression tasks.
4. A scale-sensitive feature reassembly network (SSNet) is proposed for pedestrian detection by combining the above three improvements into a whole. Extensive experiments are performed to demonstrate the effectiveness of our approach. Specifically, SSNet achieves the competitive results of 11.9% MR^−2^ and 6.3% MR^−2^ on CityPersons and Caltech datasets, respectively, which is improved by 3.7% and 4.2% compared to the baseline method.

The rest of this paper is organized as follows: Section 2 introduces the related work. Section 3 introduces the pipeline of the proposed SSNet. The multiparallel branch sampling module, context enhancement fusion module, and RoI feature reassembly module are also introduced in Section 3. Section 4 presents the experimental results related to the proposed SSNet on two benchmark datasets and verifies the effectiveness by comparisons with the baseline method and other methods. Lastly, the conclusions are drawn in Section 5.

## 2. Related Work

### 2.1. Deep Pedestrian Detection

Recently, coupled with the growth and expansion of deep learning technology [15,20,21], pedestrian detection approaches [22,23,24] have achieved impressive performance on pedestrian benchmarks [5,25]. Generally, CNN-based object detectors can be roughly divided into two types according to whether there is a procedure of extracting region proposals, i.e., two-stage approach [17,22,23,24] and single-stage approach [26,27]. The former performs a coarse-to-fine detection pipeline, which first generates a series of conceivable region proposals, and further classifies these proposals and regresses their position coordinates. The latter abandons the region proposal step and directly utilizes feed-forward CNN to predict the bounding boxes of interests. As a result, it usually has superiority in detection speed but yields lower accuracy compared with the two-stage methods.

Single-stage detector. The original single-stage detector is based on the anchor mechanism. YOLO [26] is the first single-stage detection method in the deep learning era, which divides the image into multiple regions and predicts the bounding box and class probability of each region simultaneously. SSD [27] employs different layers of the network to detect objects of various scales rather than only predicting on the top-level feature. In terms of pedestrian detection, Ren et al. [28] converge beneficial contextual information of different feature maps by a recurrent rolling convolution framework to improve single-stage detectors. ALFNet [29] proposes an asymptotic localization fitting module to progressively refine default anchor boxes based on the single-stage architecture.

In recent years, a more effective anchor-free strategy has been proposed to eliminate the hyperparameters related to anchors, which has greater generalization potential. CornerNet [30] generates objects’ top-left and bottom-right corners with the heat map and further groups them via associative embedding. CenterNet [31] adopts keypoint estimation to find the center point of an object and regresses it to an axis-aligned box. Similarly, TLL [32] devises an FCN-based network to predict the top and bottom vertexes, which are used to locate the somatic topological lines of pedestrian instances imaged at multiple scales. CSP [33] holds a new viewpoint where detecting pedestrians is regarded as a high-level semantic feature detection task and employs simplified convolutions for scale and center predictions.

Two-stage detector. Faster RCNN [17] is a typical two-stage object detection method, which utilizes a region proposal network (RPN) instead of the selective search algorithm to extract plausible proposals, thereby greatly improving the detection efficiency. A series of innovative works have emerged based on this paradigm. For instance, RPN+BF [23] appropriately modifies the RPN for handling small pedestrians and trains a cascaded boosted forest classifier to mine hard negatives. Zhang et al. [5] propose five adjustments to improve the vanilla faster RCNN. SA-Fast RCNN [34] designs a scale-aware model based on the divide-and-conquer philosophy to deal with the scale problem in pedestrian detection. SDS-RCNN [35] utilizes segmentation information to learn more robust and discriminative features. HyperLearner [36] integrates channel features such as edge, optical flow, and disparity into CNN-based pedestrian detectors. MS-CNN [22] implements multiscale detection at intermediate network layers where receptive fields adopt distinct pedestrian scales. Owing to their prominent performance on pedestrian benchmarks [5,25], we employ the two-stage framework as the backbone pipeline in this paper.

### 2.2. Detection Methods for Handling Scale Challenge

Methods based on multiple images. As one of the most critical challenges in object detection, the scale problem has attracted widespread attention. The image pyramid [8,9] is an instinctive strategy to improve detection performance. In this way, an image is scaled to different sizes and served as multiple inputs to the network. SNIP [8] proposes a novel scale normalization scheme for the image pyramid to train the instances of proper sizes in each image scale. To expedite multiscale training, SNIPER [9] only processes context regions around the ground-truth instances and samples negative chips for each scale. Nevertheless, whether in SNIP or SNIPER, the feature extraction from images of each scale is performed independently, resulting in relatively expensive computational and memory costs. Considering these limitations, image pyramid methods are not utilized as much for practical applications.

Methods based on multilevel features. Instead of increasing the number of input images, some works apply multilevel features extracted from the backbone network to remedy wide-range scale variation with less computational costs. HyperNet [37] concatenates deep and shadow features to obtain finer features for accurate prediction. FPN [10] introduces a top-down pathway and lateral connections to combine the feature maps of different spatial resolutions; thus, low-level features are instilled with more semantic information. PANet [11] further extends the FPN architecture by designing the bottom-up path and adaptive feature pooling. Libra FPN [12] integrates pyramidal features generated by FPN and refines them to learn a residual feature map. However, the above methods hardly consider the information loss of the top-level feature during feature propagation. NAS-FPN [13] uses a combination of scalable search space and a neural architecture search algorithm to automatically design feature network topology. Although NAS-FPN is flexible and performant for building the detection model, it needs thousands of GPU hours during the search, and the resulting feature network is irregular. Efficientdet [14] also leverages neural architecture search but in a more intuitive way. A weighted bidirectional feature network and a customized compound scaling method are proposed in Efficientdet to improve the accuracy and efficiency for object detection, but it still requires high computing power.

Several efforts [38,39] have demonstrated the significance of context on vision tasks. Thundernet [38] uses both global and local contexts to refine feature representation for general object detection. PSPNet [39] leverages pyramid pooling to generate global context for semantic segmentation. Inspired by these works, we apply a context enhancement module to tackle information loss of the top-level feature. Different from [38,39], adaptive average pooling is employed to generate the rich context in this paper.

Receptive Filed. Inception [40] adopts standard convolution layers with different kernels to accomplish multiple receptive fields. DCN [41] designs the deformable convolution layer to model objects of various scales but introduces more parameters. In contrast, the dilated convolution [18,42] adjusts sampling locations with the original weights, which is more economical. By combining the dilated convolution and Inception block into a single-stage detector, RFB [19] achieves good performance with less overhead. Motivated by [18,19], a lightweight sampling module is delivered to generate multiscale features. 

### 2.3. Strategy of RoI Feature Extraction

In FPN [10], each RoI is assigned to a specific feature level based on its own size. Naturally, two proposals with a slight scale difference can be allocated to different levels, leading to arbitrary allocation results. To avoid this limitation, PANet [11] proposes pooling features from all levels for each proposal and selects element-wise useful information through the max fusion operation. Nevertheless, the extra fully connected layers used to adapt proposals greatly increase the parameters. Instead, we introduce a weight-adaptive way to reassemble RoI features for better prediction. Mask RCNN [43] proposes a quantization-free technology called RoIAlign to fix the misalignment between the RoI and the extracted features, which is also adopted in this paper.

## 3. Proposed Approach

### 3.1. Overview

**Figure 3:**
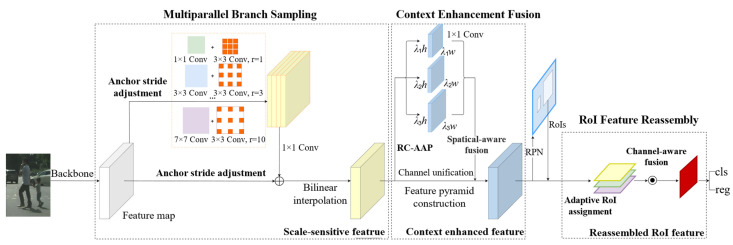
Overview of the proposed SSNet framework. The multiparallel branch sampling module is first applied to achieve adequate sampling; it consists of two parts: the flexible anchor stride adjustment scheme for capturing more small pedestrians and the modified RFB for generating multiscale features. Subsequently, the context enhancement fusion module is devised to mitigate the information loss of high-level features in channel unification. After that, the RoI feature reassembly module is used to refine proposal representations in an adaptive manner.

As illustrated in Figure 3, the proposed SSNet is mainly composed of four parts: the backbone network, multiparallel branch sampling (MBS), context enhancement fusion (CAF), and RoI feature reassembly (RFR). Considering a single image *I* as input, the backbone network is first applied on *I* to extract a series of feature maps with different resolutions, which can be defined as follows:

(1) $$\phi_{i}=f_{i}\left(\phi_{i-1}\right)=f_{i}\left(f_{i-1}\left(\ldots f_{2}\left(f_{1}\left(I\right)\right)\right)\right)$$

where $f_{i}\left(\cdot \right)$ refers to a stack of convolution or pooling layers, and $\phi_{i}$ represents the generated feature maps from the *i*th layer. $\Phi_{origin}=\left\{\phi_{1},\;\phi_{2},\ldots ,\;\phi_{n}\right\}$ are all output feature maps extracted by an n-layer backbone network. In this paper, the ResNet-50 [15] is adopted as a backbone (i.e., *n* = 5). Then, MBS is further employed to capture multi-scale features based on $\Phi_{origin}$:

(2) $$\Phi_{scale}=ℳ\left(\Phi_{origin}\right)$$

where $\Phi_{scale}$ denotes scale-sensitive features, and ℳ(·) is the multiparallel branch sampling network, which consists of the flexible anchor stride adjustment scheme and the RFB [19] block with proper modification. The top-level feature of $\Phi_{scale}$ is enhanced by CAF for better feature aggregation, resulting in $\Phi_{det}=\left\{\phi_{L}^{det},\;\phi_{L+1}^{det},\;\ldots ,\;\phi_{N}^{det}\right\}$, where 1 < *L* < *N*, and $\phi_{i}^{det}\left(i=L,\;L+1,\;\ldots N\right)$ is the pyramid feature following FPN [10].

Ultimately, these fused features are leveraged by RFR to generate high-quality RoI features for the final classification and regression tasks, which can be described as follows:

(3) $$Dets=\{𝒫\left(\Phi_{det}^{\prime },ℬ\right)=\left\{cls\left(\Phi_{det}^{\prime },ℬ\right),\;reg\left(\Phi_{det}^{\prime },ℬ\right)\right\}$$

(4) $$ℬ=N\left(\Phi_{det}\right)$$

(5) $$\Phi_{det}^{\prime }=ℛ\left(\Phi_{det},ℬ\right)$$

where N(·) and ℛ(·) represent RPN [17] and our RFR, respectively. ℬ denotes the region proposals relied on predefined anchors, and $\Phi_{det}^{\prime }$ is the reassembled RoI features. 𝒫(·) refers to the detection head that converts the features map $\Phi_{det}^{\prime }$ into detection results. Specifically, 𝒫(·) consists of two elements, i.e., cls(·) which predicts the classification confidences, and reg(·), which predicts the position offsets of the anchor boxes. Both of them are fully connected layers. The proposed detector can be trained end to end.

### 3.2. Multiparallel Branch Sampling

There are two fixed factors in the backbone network that affect the detection performance, namely the receptive field (RF) and the anchor stride (AS), which are not considered simultaneously in most methods.

Large anchor stride is an inimical issue. In the anchor-based detection scheme, AS refers to the shrinkage factor of the feature map relative to the original image. The larger anchor stride of high-level feature maps is prone to skip smaller instances directly, leading to missed detection. Consequently, an appropriate anchor stride is essential for pedestrian detection in road scenes where small-scale pedestrians dominate. Taking ResNet-50 used in our work as an example, $\Phi_{origin}=\left\{\phi_{1},\;\phi_{2},\;\phi_{3},\;\phi_{4},\;\phi_{5}\right\}$ is a set of feature maps decreased in size progressively, and their AS relative to the input image are *S_A_* = {2, 4, 8, 16, 32} pixels. In other words, the value of $S_{A}$ can only be an exponential multiple of 2, and such strides with a fixed rate in the backbone network restrict the sampling quality.

The fixed RF is another unfavorable problem. As reported by [16], the most suitable RF is strongly related to the scale of the objects, that is, a larger RF is required to detect large objects, while a smaller one is needed for small objects. However, most pedestrian detectors usually set the RF to the same size, which lacks the adaptability to pedestrians at different scales. There are some efforts that focus on adjusting the receptive field in other vision tasks, such as ASPP [42] and Inception [40], while RFB [19] combines these two methods from the perspective of the human visual cortex and achieves better detection performance. Inspired by this, we employ RFB with several changes as part of our module to model pedestrians at different scales. 

**Figure 4:**
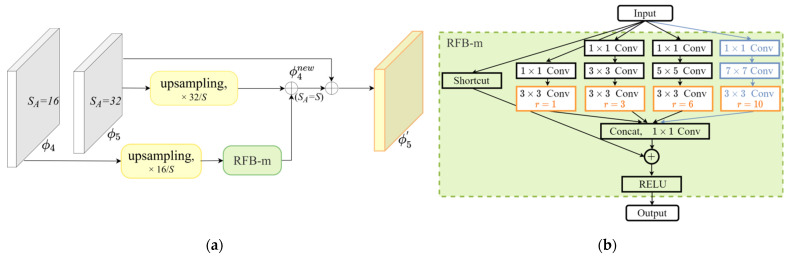
Illustration of the proposed multiparallel branch sampling module. (**a**) Architecture of MBS, fully considering the sampling stride and adaptability to different pedestrian scales; (**b**) inner structure of modified RFB.

The proposed multiparallel branch sampling (MBS) module is illustrated in Figure 4a. It includes two parts, i.e., the anchor stride adjustment scheme and the modified RFB block, denoted as RFB-m. The value of *S_A_* is flexibly controlled by changing the size of the feature map, allowing more adaptive sampling. Only $\phi_{4}$ and $\phi_{5}$ are used for fusion with a view toward reducing the network parameters, while other less relevant feature layers are ignored. The first channel of MBS upsamples the feature map $\phi_{4}$ to make its *S_A_* = *S*, where *S* represents the expected anchor stride. Then, the upsampled $\phi_{4}$ is fed into RFB-m to obtain different receptive fields, which capture the characteristic information of diverse pedestrians. On the second channel, $\phi_{5}$ is also upsampled to the uniform size. Eventually, a scale-sensitive feature map $\phi_{4}^{new}$ is generated by the element-wise addition of the two channels. Considering that too large of a dilation rate may lead to sparse sampling, the $\phi_{4}^{new}$ is further fused with the original feature $\phi_{5}$. Mathematically, the MBS module can be formulated as follows:

(6) $$\phi_{4}^{new}=T\left(U\left(\phi_{4}\right)\right)\;⊕\;U\left(\phi_{5}\right)$$

(7) $$\phi_{5}^{\prime }=U\left(\phi_{4}^{new}\right)\;⊕\;\phi_{5}$$

where U(·) and T(·) represent the upsampling operation and RFB-m, respectively. Bilinear interpolation is adopted in our implementation, where the scale factor is *S_A_*/*S*. RFB-m performs sampling with an expected stride *S*. $\phi_{5}^{\prime }$ is the final feature map generated by MBS module. 

As shown in Figure 4b, RFB-m shares a similar structure with RFB. It mainly contains two components: the standard convolution layers with different kernels and the dilated convolution layers with various dilated rates. It establishes the correlation between different convolution kernels and the dilated rates to effectively adjust the receptive field. Specifically, the modification we make includes two aspects. First, we add one more branch with a larger kernel and dilated rate to capture more features, namely a 7 × 7 convolution layer and a dilated convolution with a dilated rate of 10. Second, as [18] reported that dilated rates within a group should not have a common factor relationship (like 2,4,8, etc.) for better results, we simply constrain the dilation rates of all branches as follows:

(8) $$D\left(r_{1},r_{2},\ldots r_{n}\right)=1$$

where $\left[r_{1},\ldots \ldots r_{n}\right]$ denotes a set of dilated rates of n branches in ascending order, and D refers to the max common divisor. Then, the convolution kernels and dilated rates are set to [1 × 1, 3 × 3, 5 × 5, 7 × 7] and [1, 3, 6, 10], respectively. For the purpose of reducing the computational overhead and increasing nonlinear layers, the 5 × 5 and 7 × 7 conv-layers are replaced by two and three consecutive 3 × 3 conv-layers in the actual implementation, respectively. Finally, the feature maps of four branches are concatenated and passed through a 1 × 1 conv-layer to create a new convolution array.

With the help of MBS, we have achieved subsampling in the backbone network. We discover that the optimal S depends on the distribution of small-scale pedestrians in the dataset. In order to make a trade-off between detection accuracy and training overhead, the value of S is set to 18, which will be described in detail in Section 4.3.2.

### 3.3. Context Enhancement Fusion 

**Figure 5:**
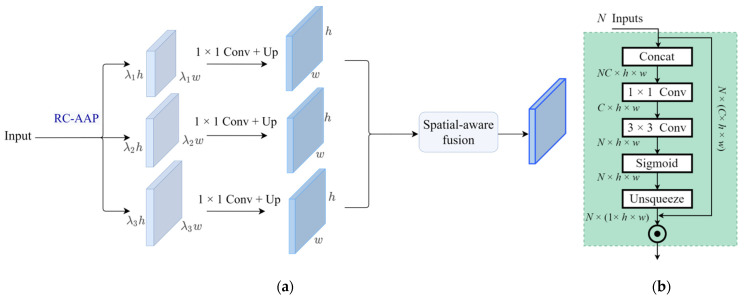
Illustration of context enhancement fusion module. (**a**) The process of generating and fusing different context features, where RC-AAP denotes ratio-constant adaptive average pooling, ‘Up’ means an upsampling operation; (**b**) the details of spatial-aware fusion.

Following FPN, context enhancement fusion (CAF) aims to aggregate multiscale features from the backbone effectively. Formally, given a series of features with various resolution $\Phi_{scale}=\left\{\phi_{2},\;\phi_{3},\;\phi_{3},\phi_{5}^{\prime }\right\}$, where $\phi_{i}$ denotes a feature level of index *i*, $\phi_{1}$ is ignored to save memory. Our goal is to find a transformation ℱ(·) that can combine $\Phi_{scale}$ properly and create the feature hierarchy $\Phi_{det}=\left\{\phi_{L}^{det},\;\phi_{L+1}^{det},\;\ldots ,\;\phi_{N}^{det}\right\}$ that is responsible for the detection task. In FPN, $\Phi_{scale}$ is first fed into a 1×1 convolution layer to achieve channel unification. We denote the output feature maps as $M=\left\{m_{2},\;m_{3},m_{4},\;m_{5}\right\}$, then these features are fused in a top-down manner:

(9) $$\phi_{L}^{det}=\{\begin{array}{l} \alpha \left(m_{L}\right),\;\;\;\;\;\;L=n\\ \alpha \left(m_{L}+𝓇(m_{L+1})\right),\;L\in \left[2,n-1\right]\;\;\; \end{array}$$

where 𝓇 is an operation for resolution matching, and α is the weight of a 3 × 3 convolutional layer. Under this strategy, the shallow features are progressively enhanced by the semantics of deep features, which indicates that the top-level feature is extremely important. However, $m_{5}$ struggles with information loss due to channel dimension reduction. To address this problem, the proposed CAF mainly consists of two parts, spatial context generation (SCG) and spatial-aware fusion (SAF), shown in Figure 5.

SCG focuses on producing multiple context feature maps with different sizes. As illustrated in Figure 5a, the adaptive average pooling with the constant ratio is first applied to $\phi_{5}^{\prime }$ to construct features containing rich context information, which can be expressed as $\Psi_{c}=\left\{\lambda_{1}\times R,\lambda_{2}\times R,\ldots \lambda_{n}\times R\right\}$, where *R* represents the feature resolution of $\phi_{5}^{\prime }$, and $\left\{\lambda_{1},\ldots ,\lambda_{n}\right\}$ are the constant ratios that we set to $\left\{\lambda_{1}=0.1,\lambda_{2}=0.2,\lambda_{3}=0.3\right\}$ by default. After this step, $\Psi_{c}$ undergoes a 1 × 1 convolution layer to reduce the channel number to 256. Ultimately, these features are extended to the scale of *R* through bilinear interpolation for later fusion. We further devise SAF to dig the importance of each input contextual feature and fuse them through learnable weights rather than a simple summation. The detailed structure of SAF is shown in Figure 5b. To be specific, taking the upsampled features as input, a weight map is generated for each feature by a combination of the concatenation operation and convolutional layers. With the help of spatial weights, the context features are aggregated into $m_{6}$, which encodes multiscale context information.

For the purpose of enhancing the top-level feature, *m*_6_ is combined with *m*_5_ by element-wise addition. After that, the information from high-level features is propagated in a top-down manner, and a feature pyramid $\Phi_{det}=\left\{\phi_{L}^{det},\;\phi_{L+1}^{det},\;\ldots ,\;\phi_{N}^{det}\right\}$ is constructed by 3 × 3 convolution layers. The final output feature has a similar structure to FPN but accomplishes better performance on pedestrians imaged at multiple scales.

### 3.4. RoI Feature Reassembly

In the detection pipeline, proposal refinement is performed based on the fused feature hierarchy $\Phi_{det}$. To this end, FPN adopts a heuristic strategy to assign an RoI of width w and height *h* to a specific pyramid level, which can be formulated as

(10) $$k=\lfloor k_{0}+\log_{2}\left(\sqrt{wh}/224)\right\rfloor$$

where $k_{0}$ is the target level corresponding to an RoI with $w\times h=224^{2}$. Intuitively, each RoI is allocated according to its own size. If an RoI is smaller than $224^{2}$, then it should be mapped into a lower level, while a larger one should be assigned to a higher level. Nevertheless, this scheme could lead to suboptimal results due to equivocal choices, which becomes worse under the dilemma of the small pedestrian relative scale (PRS) mentioned in Section 1. Considering this defect, PANet [11] assigns each RoI to all pyramid levels and performs max fusion to aggregate RoI features after adapting them with fully connected layers. Although this method improves the performance, the max operation it uses ignores those features with a lower response, which may be useful for subsequent tasks. Moreover, extra fully connected layers significantly increase the network parameters.

**Figure 6:**
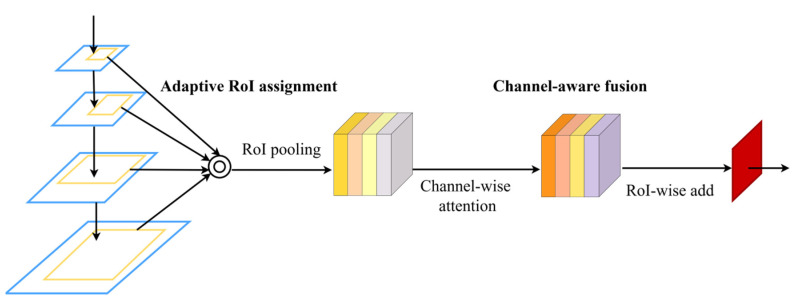
Illustration of the proposed RoI feature reassembly module. Different from the heuristic strategy, RFR assigns each RoI to all feature levels and reassembles RoI features from the perspective of channel importance.

Based on the above analysis, a novel RoI feature reassembly (RFR) module is proposed to fully utilize features in multiple levels. As demonstrated in Figure 6, each RoI is first mapped into all levels following PANet, as denoted by the light-yellow regions in Figure 6. Instead of treating all region features equally without distinction, we adopt a channel-aware fusion (CAF) method to generate weight maps for each input level. It is inspired by SENet [44] but with a different goal, which is to reassemble RoI features according to channel importance. CAF introduces fewer parameters compared with the fully connected layers utilized in PANet. In particular, the height vs. width ratios of anchors are modified as {1∶1, 1.5∶1, 2∶1, 2.5∶1, 3∶1} for RPN in consideration of the human body shape. Thanks to RFR, we avoid the arbitrary allocation strategy and improve RoI features, which is beneficial for both training and inference phases.

### 3.5. Training Objective

Finally, the reassembled RoI features go through a detection head to produce the classification score and the position coordinates for each proposal. Since every component in our framework is differentiable, the proposed method can be trained end to end with following multitask loss function:

(11) $$L\left(\left\{p_{i}\right\},\left\{t_{i}\right\}\right)=\frac{1}{N_{cls}}\overset{N_{cls}}{\underset{i=1}{\sum }}L_{cls}\left(p_{i},c_{i}^{*}\right)+\frac{\xi }{N_{reg}}\overset{N_{reg}}{\underset{i=1}{\sum }}I_{\left[c_{i}^{*}>0\right]}L_{reg}\left(t_{i},g_{i}^{*}\right)$$

where i is the index of samples, *N_cls_* is the total number of samples, and *N_reg_* is the number of positive samples. *L_cls_* and *L_reg_* represent the classification loss and bounding box regression loss, respectively. The prediction confidence and ground-truth class label for each training RoI are denoted as $p_{i}$ and $c_{i}^{*}$. *t_i_* is a vector representing the 4D parameterized coordinates of the predicted bounding box, and $g_{i}^{*}$ refers to the corresponding bounding box regression target. $I_{\left[c_{i}^{*}>0\right]}$ is an indicator function, being 1 when $c_{i}^{*}>0$ and 0 otherwise. This means that the *L_reg_* of a background RoI whose class label is $c_{i}^{*}=0$ will be ignored. The hyperparameter *ξ* is used for tuning the weight between multitask losses. All experiments use *ξ* = 1. For classification loss *L_cls_*, we use cross-entropy loss over two classes (pedestrian vs. not pedestrian). For bounding box regression loss *L_reg_*, we adopt *SmoothL*1 Loss [45].

## 4. Experiments

### 4.1. Datasets and Evaluation Metric

We evaluate our approach on two pedestrian detection benchmark datasets: Caltech-USA [25] and CityPersons [5]. 

#### 4.1.1. Caltech-USA Dataset

A 2.5-h autonomous driving video is divided into 11 sets in the Caltech-USA dataset. The first six sets (set00-set05) are used for training, and the last five sets (set06-set10) correspond to testing. We adopt the Caltech 10× training set [46], as widely done in [5,23,24,32,47], which provides refined annotations by combining automatic and manual reannotation. We evaluate our models with the standard testing set using new annotations provided by [46].

Caltech-USA annotates an occluded pedestrian with two bounding boxes that denote the visible and full pedestrian extent. The fraction of occlusion is calculated as 1 minus the visibility ratio, which is computed as visible pedestrian area divided by total pedestrian area. The reasonable subset is a widely used subset for evaluating pedestrian detectors. It refers to pedestrians whose height is greater than 50 pixels and visibility ratio is greater than 65%. 

#### 4.1.2. CityPersons Dataset

CityPersons is a more challenging predominant dataset for pedestrian detection with a large diversity. It consists of about 35,000 persons with meticulously labeled bounding boxes and about 13,000 ignored region annotations. The definition of the reasonable subset is the same as Caltech. The small, middle, and large subsets correspond to pedestrians with height ranges of [50,75], [75,100], and [100,∞], respectively. 

For fair comparison, we use the official training set with 2975 images and the validation set with 500 images to train and test the proposed model, respectively.

#### 4.1.3. Evaluation Metrics

For evaluation, we follow the standard Caltech evaluation metric [25], which is the average-log miss rate (MR) computed over the false positive per image (FPPI) range of [10^−2^, 10^0^], denoted as MR^−2^. Lower is better.

### 4.2. Implementation Details

All networks are trained on four GPUs (NVIDIA GTX 1080Ti). Stochastic gradient descent (SGD) with 0.9 momentum and 0.0001 weight decay is used to optimize the networks. We conduct all experiments on MMDetection (OpenMMLab detection toolbox and benchmark). ResNet-50 pretrained on ImageNet [6] is the backbone. We adopt faster RCNN with an FPN neck as the baseline. 

For the CityPersons dataset, the base learning rate is set to 0.01 for the first 8 epochs and further decreased to 0.001 for the last 4 epochs. For the Caltech dataset, we also start with the initial learning rate of 0.01 and decay it by a factor of 10 after 7 epochs with a total of 10 epochs. A minibatch comprises two images per GPU, except for CityPersons where a minibatch involves only one image due to the physical limitation of the GPU memory. Unless otherwise specified, the settings of other hyperparameters follow the defaults from MMDetection and are strictly consistent in all experiments. In the inference phases, we measure the time cost of various methods on a single GPU.

### 4.3. Ablation Study

#### 4.3.1. Baseline Comparison

Ablation experiments are conducted on the CityPersons validation set to demonstrate the effectiveness of each proposed component in SSNet, and all results are shown in Table 1. Consistent with the reasonable evaluation protocol [5], only the reasonable subset of pedestrians is used for training. Multiparallel branch sampling, context enhancement fusion, and RoI feature reassembly are gradually applied to the baseline method. We also present the gains brought by the combination of different components, which shows that the three components are complementary to each other.

Multiparallel Branch Sampling. To remedy the large-scale variation, we introduce a multi-parallel branch sampling (MBS) module into the backbone network, noted as Method(b) in Table 1. It achieves an improvement of 1.6% in reasonable MR^−2^ compared to the baseline (from 15.6% to 14.0%). The gains of MR^−2^ on each scale (small, middle, large) are 2.6%, 1.2%, and 1.7%, respectively, which means our MBS effectively captures pedestrian information at different scales. It is worth noting that MBS tends to improve the detection performance on small-scale pedestrians as designed, indicating that the flexible anchor stride can sample more small samples and achieves the expected effect.

Context Enhancement Fusion. The detector combined with the context enhancement fusion (CEF) module reduces the error on the reasonable subset from 15.6% to 13.7%, noted as Method(c) in Table 1. It can be seen that CEF is of much help for small- and middle-scale pedestrians with 18.0% and 7.4% MR^−2^, respectively. Meanwhile, the MR^−2^ of large-scale pedestrians is also reduced by 1.4% to 7.6%. These results show that the additional context information injected into the top-level feature map *m*_5_ significantly alleviates the information loss during compressing channel dimensions. In particular, spatial-aware fusion aggregates contextual features by learning the importance of different feature maps and improves the representation ability of the feature pyramid with less computational cost.

RoI Feature Reassembly. According to the experiment of Method(d) in Table 1, the RoI feature reassembly (RFR) module improves the reasonable MR^−2^ from 15.6% to 14.1%, and the MR^−2^ improvements of small and middle-scale pedestrians contribute most to the final performance improvement, namely 2.5% and 1.7%, respectively. Considering the distribution of pedestrian sizes in natural images where small and middle scales dominate, these results further demonstrate the effectiveness of our RFR strategy. In the traditional RoI allocation scheme, each RoI is allocated to a specific feature layer, ignoring the possible contribution of other feature layers. RFR fully absorbs the effective information of each pyramid layer through the reassembly method and obtains high-quality RoI features, which is conducive to subsequent classification and regression tasks.

We further combine any two of the above three modules, noted as Method(e), (f), and (g) in Table 1. Compared with the baseline detector, these approaches reduce the MR^−^^2^ by 2.4% (from 15.6% to 13.2%), 2.2% (from 15.6% to 13.4%), and 2.3% (from 15.6% to 13.3%) on the reasonable subset, respectively, achieving more gains than applying a single component. This shows that these modules are complementary to each other. Method(e) has the largest improvement for small-scale pedestrians by 3.9%, indicating that MBS and CEF cooperate well in small pedestrian detection. In addition, Method(g) equipped with RFR and CEF have more improvements for middle- and large-scale pedestrians. This may benefit from more information of spatial details in the lower layers, which is helpful for location.

**Figure 7:**
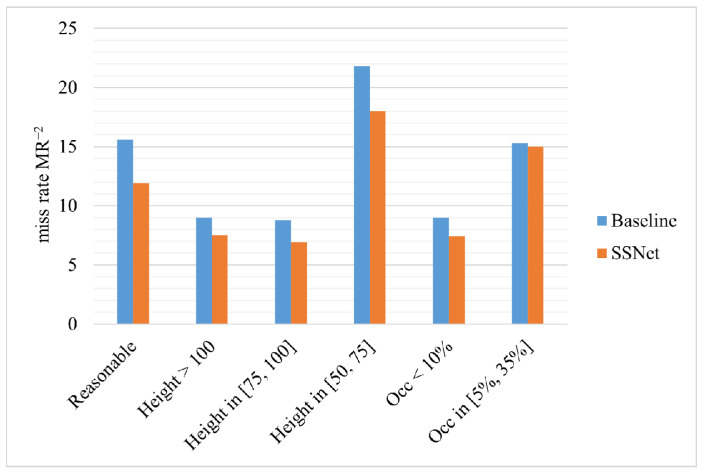
Detection quality (average-log miss rate) for different settings of the test data. Each group shows the baseline and SSNet detectors.

Finally, we integrate the three modules into the baseline together, noted as Method(h) in Table 1. It can be seen that SSNet achieves an absolute reduction of 3.7% (from 15.6% to 11.9%) on the reasonable subset. Figure 7 depicts the detection quality of the baseline and SSNet for different settings of the test data. The results of SSNet on pedestrians of different heights are consistently improved, indicating that our method effectively tackles scale problems. In particular, pedestrians with a height range of [50,75] have the greatest gains, which proves that small-scale pedestrians are indeed the bottleneck of detection performance. Furthermore, SSNet boosts more on pedestrians with an occlusion fraction of less than 10%.

**Figure 8:**
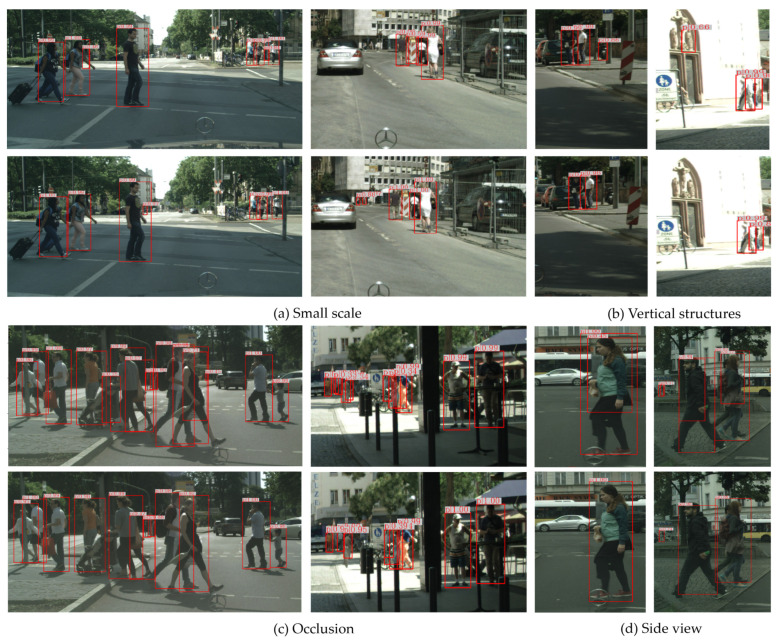
Visual comparison of the baseline method (the first and third rows) and our SSNet (the second and fourth rows) under different scenarios of the CityPersons validation set.

The visualized comparison between the baseline method and our SSNet in different situations is shown in Figure 8. The first and third rows show the results on images with the baseline method, and the second and fourth rows show the results on images with our SSNet. Obviously, SSNet is able to detect the small-scale pedestrians, which are missed in the baseline detector. There are some background errors caused by vertical structures for the baseline method, while SSNet performs well in the vertical context, shown in Figure 8b. In the occlusion scenario, SSNet is more robust than the baseline method. In addition, SSNet achieves more accurate detection and reduces false alarms to some extent for images with side-view persons.

#### 4.3.2. Multiparallel Branch Sampling

We first analyze the impact of varying *S_A_*. The experimental results and training cost on the reasonable subset of CityPersons with different sampling strides are shown in Table 2. MBS achieves the best result of 13.7% when *S_A_ =* 14. The detection performance gradually decreases as *S_A_* increases. Meanwhile, the average training time per image is reduced from 2.01 s to 1.59 s. A smaller *S_A_* is conducive to sufficient sampling. However, continuously reducing *S_A_* is not an ideal choice. As can be noticed, dense sampling will increase training overhead. We find that the optimal *S_A_* depends on the distribution of pedestrian scales in the dataset, especially on small-scale pedestrians. Considering the tradeoff between detection performance and training overhead, we set *S_A_* to 18 by default.

To demonstrate the effectiveness of our MBS module, the performance of three sampling strategies is compared in Table 3. DCN [41] and ASPP [42] adjust the receptive field in different ways, while single-scale is the baseline without any components. It can be seen that the three methods consistently reduced the reasonable MR^−2^ but slightly increased the inference time. In particular, MBS surpasses DCN and ASPP by 0.7% and 0.4%, respectively, and the improvements of MR^−2^ on small-, middle-, large-scale pedestrians are the largest among all methods. Although DCN and ASPP have improved the results to some extent, the sampling stride is still inflexible and cannot achieve the best detection performance. Moreover, the average inference time per image in MBS is less than ASPP, indicating that MBS can effectively extract scale-sensitive features with less computational overhead. 

#### 4.3.3. Context Enhancement Fusion

The importance of adaptive average pooling with constant ratios is explored in Table 4. We choose two types of global pooling to conduct experiments, namely global max pooling (GMP) and global average pooling (GAP). As can be observed, GAP achieves a better result with an absolute reduction of 0.9% MR^−2^ over the baseline, while GMP degrades the performance instead. This indicates that average pooling is more robust than max pooling, as the latter may be susceptible to peak noise. Based on this observation, GAP is replaced by ratio-constant adaptive average pooling (RC-AAP), as shown in Table 4, (d). We set λ with three values of 0.1, 0.2, and 0.3. Considering that there is only one branch in both Experiment (b) and (c), we adopt sum fusion for fair comparison. RC-AAP improves the baseline and GAP by 1.4% and 0.5%, respectively, validating the effectiveness of the diverse context provided by SCG. In addition, we combine spatial-aware fusion (SAF) with RC-AAP (Table 4, (e)) and achieve a better MR^−2^ of 13.7%.

We further study the influence of **λ** on detection performance, as shown in Table 5. The MR^−2^ decreases from 14.2% to 13.6% as the number of **λ** increases (Table 5, (a)~(d)). However, it can be noticed that the four values of **λ** do not bring more gain than the three values. Therefore, considering the tradeoff between complexity and performance, three values of **λ** are adopted by default. Meanwhile, the effects of different **λ** values are discussed in Table 5, (e)~(f). We find that when the value of **λ** changes, the performance of CEF stabilizes at 14.1% MR^−^^2^ and shows no more improvement.

#### 4.3.4. RoI Feature Reassembly

In this section, we investigate the effect of channel-aware fusion (CAF), which is inspired by SENet [44] but with a different goal of improving RoI features according to channel importance. Table 6 shows the results of three different fusion ways, including sum fusion, max fusion, and CAF, respectively. Sum fusion is slightly inferior to max fusion, as the former obtains 14.8% MR^−2^, while the latter is 14.7% MR^−2^. As expected, CAF achieves the best results with an improvement of 1.5% over the baseline on the reasonable subset. With the help of CAF, the detector absorbs the beneficial feature of other pyramid layers with less extra computation compared to PANet [11].

### 4.4. Comparisons with Other Methods on CityPersons

Here, the proposed SSNet is compared with several state-of-art methods on the CityPersons dataset, including ATT-vbb [47], FRCNN [5], RepLoss [24], TLL [32], TLL+MRF [32], OR-CNN [48], and ALFNet [29]. The results are shown in Table 7. All results of comparison methods are reported by their respective papers, and “-” means that the result is not reported by the corresponding paper. For a fair comparison, only those methods adopting subset partition criterion in [5] and feeding images with the original size as inputs are listed. Our SSNet achieves the best performance among these methods with 11.9% MR^−2^ on the reasonable subset. By referring to the results of small, middle and large subsets, the gains mainly come from the small-scale subset (+3.8%), which proves that our SSNet has advantages in small-scale pedestrian detection. The TLL approach aiming at multiscale pedestrian detection with a somatic topological line obtains 15.5% MR^−2^ on the reasonable subset, and the one with a postprocessing scheme based on MRF achieves 14.4% MR^−2^. Our SSNet surpasses the latter by 2.5%. ALFNet yields the best results for pedestrians of middle and large scales, while SSNet performs better for the reasonable and small subsets. In terms of inference speed, SSNet is faster than ALFNet with 0.20 s per image on the same running environment.

### 4.5. Comparisons with Other Methods on Caltech

To further verify the robustness of our proposed SSNet, we also conduct experiments on the Caltech dataset, which is a popular benchmark for pedestrian detection. We compare SSNet with some representative methods, including RPN+BF [23], SA-Fast RCNN [34], MS-CNN [22], UDN+SS [49], ADM [50], FRCN+A+DT [51], TLL [32], SDS-RCNN [35], and repulsion loss [24]. Table 8 reports the results on the reasonable subset. Thanks to the three components, our SSNet delivers competitive performance among other methods with an MR^−2^ of 6.3%. It also surpasses the baseline by 4.2%. The MS-CNN, SA-Fast RCNN, and TLL methods employ different technologies but have the same goal as we do, which is to remedy the large-scale variation. According to Rows 3,4,8 in Table 7, SSNet outperforms all three methods by 3.6%, 3.3%, and 1.1%, respectively, validating the superiority of SSNet for multiscale pedestrian detection. However, there is a gap of 2.3% between our SSNet and the repulsion loss method. One possible reason is that repulsion loss introduces extra powerful features, such as a supervisor. Moreover, the extra training data it uses also contributes to this result, while our detector is directly trained on the Caltech dataset. In general, the comparisons actively demonstrate the effectiveness and generalization of our method.

## 5. Conclusions

In this paper, we propose SSNet, a scale-sensitive feature reassembly network, for handling severe scale challenges in pedestrian detection under road scenes. SSNet is designed to include three modules. MBS, which considers the receptive field and the anchor stride simultaneously, is first proposed to achieve adequate sampling for pedestrians at varying scales. For subsequent multiscale feature fusion, CEF is devised to mitigate the information loss of top-level features and construct a discriminative feature pyramid. Furthermore, RFR is introduced to generate robust RoI features in an adaptive manner, which benefits both classification and regression tasks. Note that these modules are lightweight and can be trained end to end. Experiments on the Caltech and CityPersons datasets demonstrate the effectiveness of our approach, especially for small-scale pedestrians.

## Figures and Tables

**Table 1:** Ablation evaluation of the proposed SSNet on CityPersons validation set (MR^−^^2^/%).

Methods	(a)	(b)	(c)	(d)	(e)	(f)	(g)	(h)SSNet
Multiparallel branch sampling?		✓			✓	✓		✓
Context enhancement fusion?			✓		✓		✓	✓
RoI feature reassembly?				✓		✓	✓	✓
Reasonable	15.6	14.0	13.7	14.1	13.2	13.4	13.3	11.9(+3.7)
Small	21.8	19.2	18.0	19.3	17.9	18.5	18.4	18.0(+3.8)
Middle	8.8	7.6	7.4	7.1	8.2	7.2	7.3	6.9(+1.9)
Large	9.0	7.3	7.6	8.0	8.3	7.4	7.1	7.5(+1.5)

**Table 2:** The miss rate and average training overhead per image on CityPersons validation set with varying *S_A_* (MR^−2^/%).

Anchor Stride *S_A_*	14	16	18	20	22	24
Reasonable	13.7	13.9	14.0	14.4	14.3	14.9
Training time per image	2.01 s	1.95 s	1.89 s	1.79 s	1.67 s	1.59 s

**Table 3:** Comparison of MBS with other sampling strategies on CityPersons validation set (MR^−2^/%).

Methods	Single-Scale	MBS	DCN	ASPP
Reasonable	15.6	14.0	14.7	14.4
Small	21.8	19.2	20.1	20.5
Middle	8.8	7.6	7.6	8.0
Large	9.0	7.3	8.7	7.8
Inference time per image	134 ms	146 ms	156 ms	140 ms

**Table 4:** Comparison of different pooling methods. GMP, GAP, RC-AAP mean global max pooling, global average pooling, and ratio-constant adaptive average pooling, respectively. SAF means spatial-aware fusion (MR^−^^2^/%).

Methods	Pooling Type	*λ*	Reasonable
(a)	Baseline	-	15.6
(b)	GMP	-	16.4
(c)	GAP	-	14.7
(d)	RC-AAP w/o SAF	0.1,0.2,0.3	14.2
(e)	RC-AAP w/ SAF	0.1,0.2,0.3	13.7

**Table 5:** Results on CityPersons validation set using varying λ settings. SAF means spatial-aware fusion (MR^−^^2^/%).

Methods	Pooling Type	*λ*	Reasonable
(a)	RC-AAP w/SAF	0.1	14.2
(b)		0.1,0.2	14.1
(c)		0.1,0.2,0.3	13.7
(d)		0.1,0.2,0.3,0.4	13.6
(e)		0.1,0.2,0.4	14.1
(f)		0.1,0.2,0.6	14.1

**Table 6:** Ablation study of the RoI feature reassembly module with different fusion ways. Note that CAF is a channel-wise attention strategy inspired by SENet (MR^−^^2^/%).

Methods	Reasonable	Small	Middle	Large
Baseline	15.6	21.8	8.8	9.0
Sum	14.8	18.2	8.1	8.7
Max	14.7	20.8	8.0	7.7
CAF	14.1	19.3	7.1	8.0

**Table 7:** Comparison of the proposed SSNet with other methods on CityPersons validation dataset. Results test on the original image size (1024 × 2048 pixels) are reported. Note that “-” means the result is not reported by the corresponding paper (MR^−^^2^/%).

Methods	Backbone	Reasonable	Small	Middle	Large	Test Time
ATT-vbb	VGG-16	16.4	-	-	-	-
FRCNN	VGG-16	15.4	25.6	7.2	7.9	-
FRCNN+Seg	VGG-16	14.8	22.6	6.7	8.0	-
RepLoss	ResNet-50	13.2	-	-	-	-
TLL	ResNet-50	15.5	-	-	-	-
TLL+MRF	ResNet-50	14.4	-	-	-	-
OR-CNN	VGG-16	12.8	-	-	-	-
ALFNet	ResNet-50	12.0	19.0	5.7	6.6	0.27 s/img
SSNet (ours)	ResNet-50	11.9	18.0	6.9	7.5	0.20 s/img

**Table 8:** Comparison of the proposed SSNet with other methods on the standard test set of Caltech dataset (MR^−^^2^/%).

Methods	Backbone	Reasonable
RPN + BF	VGG-16	9.5
SA-Fast RCNN	VGG-16	9.6
MS-CNN	VGG-16	9.9
UDN + SS	VGG-16	11.5
ADM	ResNet-50	8.6
FRCN + A + DT	VGG-16	8.0
TLL	ResNet-50	7.4
SDS-RCNN	VGG-16	7.3
Repulsion Loss	ResNet-50	4.0
SSNet (ours)	ResNet-50	6.3

## Data Availability

The data presented in this study are available on request from the author. The data are not publicly available due to involving a certain degree of privacy.
